# District Level Geospatial Analysis of Utilization of ICDS Services Among Children in India

**DOI:** 10.3389/fpubh.2022.874104

**Published:** 2022-07-01

**Authors:** Pradeep Kumar, Sampurna Kundu, Rahul Bawankule

**Affiliations:** ^1^Department of Survey Research and Data Analytics, International Institute for Population Sciences, Mumbai, India; ^2^Centre of Social Medicine and Community Health, Jawaharlal Nehru University, New Delhi, India; ^3^Department of Biostatistics, MGM Institute of Health Sciences, Mumbai, India

**Keywords:** non-utilization of ICDS, geospatial, district, children, Integrated Child Development Scheme (ICDS)

## Abstract

**Introduction:**

Integrated Child Developmental Services (ICDS) is the most extensive government-run health program for children with its foot spread across the complete Indian Territory. ICDS Scheme, has been provided for 40 years and has been successful in some ways. The program in reducing the undernourishment among children over the past decade has been modest and slow in India than what has been reached in other countries with comparable socio-economic measure. Therefore, this study aims to identify the district level clustering of the utilization of ICDS services in India, and the present research also tried to relate it with socio-economic and demographic factors.

**Materials and Methods:**

The data from the fourth round of the National Family Health Survey (NFHS-4) conducted in 2015–16 in India is used to carry out the analysis. We classified the country in 640 districts and employed geospatial techniques like Moran's I, univariate and bivariate local indicators of spatial association (LISA), and spatial error regression.

**Results:**

The non-utilization under ICDS scheme varied between 93% in West Siang district of Arunachal Pradesh and around 7% in the Kandhamal district of Odisha in 2015–16 in India. The univariate LISA results suggest striking geographic clustering of utilization of ICDS services among children in India (Moran's I: 0.612). On another hand, there were regions with substantially low-low clustering of non-utilization of ICDS services in southeast India, including districts in Andhra Pradesh, Chhattisgarh, Southern Madhya Pradesh, Odisha, Telangana, and West Bengal. The findings also suggest that the proportion of the rural population (−0.190), and poor households (−0.132) in the district were significantly and negatively related while the proportion of uneducated women (0.450) was positively related to the non-utilization of ICDS services within the district.

**Conclusion:**

This is the first-ever study that examined the complex interplay of the rural population, female illiteracy, poverty, SC/ST population, and Hindu population with non-utilization of ICDS services among children in the district in India. The study highlights the inter-district geographical disparities in the non-utilization of ICDS services. Further, it confirms that underprivileged districts in terms of the rural population and poor households are also disadvantageous in the utilization of ICDS services.

## Introduction

Integrated Child Development Services (ICDS) in India is the world's largest integrated early childhood program, with over more than 1.3 million centers nationwide which was launched in 1975 as a part of Human resource development to improve children's health and quality of life (1). It targets a range of interventions for early childhood care and development encompassing integrated services for the development of children below 6 years, expectant and nursing mothers and adolescent girls living in the most backward, rural, urban and tribal areas (2). The program is implemented through a network of community-level *Anganwadi* centers. ICDS scheme not only plays an important role in improving health, nutrition, and educational services for children from birth to 6 years of age but also crucial for bringing the behavioral changes in supporting care practices. The program has a significant impact on improving the survival rate, health and nutritional status, and educational outcomes of children. The ICDS population had a lower infant mortality rate and a better nutrition status than the general child population, which provides further evidence of the efficacy of the program (3). In India, 66% of the total population comprises of mother and children who are the major consumers of health services (4); hence, they are the priority, special risk, and vulnerable group. It is essential to improve the health status of the most vulnerable groups of the society. The lives of children and women are the true indicators of the strength of communities and nations.

Earlier literature reported an association of improved nutritional status and immunization status of children <3 years age, with ICDS services (5, 6). A community-based cross-sectional study in the urban area of Agartala, Tripura, revealed that three-fourth of the children were utilizing any of the services provided under the ICDS scheme. Further, the study cited, more than half of the non-utilizers reported that they send their children to private nursery schools where 42.3% of parents did not know about the services for children below 3 years (7). Another study done in 40 Anganwadi of Bhopal city found that not every third beneficiary in the 10th was satisfied with the services (8). According to Chudasama et al. (9) did a study, in Gujarat reported 48.3% coverage among children of supplementary nutrition and found program gaps in coverage of supplementary nutrition in children (9). The research found that pregnant women living in ICDS areas were more likely to seek ANC and immunizations than those who were not living in ICDS areas (3).

A community based cross-sectional study on urban slum reported 77.48% utilization ICDS services. The study also found a strong significant association between socio-economic status and utilization of ICDS services. The utilization of services was less among families those belonged to more Per-Capita Income group. Therefore, an inverse relation can be seen between SES and utilization of ICDS services (10). The lack of awareness, regarding services, household work or outdoor work and longer distance from anganwadi was the major reason for non-utilization of regular usage of ICDS services (10, 11). Among rural women who received nutrition and health-education specifically from the ICDS had, 12.3% higher institutional delivery compared to those who did not receive (12). In addition, the presence of ICDS centers in communities helps in reducing child stunting (13). A study done by Dutta and Ghosh (14) found impact of receiving supplementary feeding was insignificant on child stunting measures, though the program can break the intractable barriers of child stunting only when the child successfully receives not only just the supplementary feeding but also his caregiver collects crucial information on nutritional awareness and growth trajectory of the child (14).

Davis et al. (15), found factors associated with accessing ICDS services among women in Rajasthan, India. Households that accessed ICDS services were more likely to report receiving nutrition information from ICDS centers, to purchase ORS in the last year, and to give oral rehydration solution (ORS) to children who had diarrhea. Women who decide how much food to serve each family member or spend money without discussing it first with someone else were more likely to receive benefits from ICDS centers. Those who spoke with their spouse about household nutrition needs were less likely to report accessing ICDS services (15). Other factors such as wealth of the household, maternal education and caste showed the largest positive associations with use of ICDS services. However, utilization of ICDS services varied at the sub-national level. Although, overall use of ICDS services had improved and reached marginalized groups such as disadvantaged castes and tribes, the poorest quintiles of the population were still left behind, especially in the largest states that carry the highest burden of undernutrition (16). Although, few literature reported underutilization of ICDS scheme by the community (17, 18).

Despite almost 40 years of implementation of ICDS, nearly half of the children aged below 6 years are not benefited/utilized one or more services (food supplements, immunizations, health check-ups, early childhood care/preschool, and weighing service) from AWCs (19). A recent study by Jawahar and Raddi (20), noted that children under 5 years were not utilizing services fully due to the mother's lack of knowledge. Mothers are the primary care providers and the knowledge about the services provided by ICDS are necessary. In several regions due to lack of awareness the rate of utilization is more (20, 21). Around 16% children are low birth weight (16.4%) (22), and about three-fifth of children are fully immunized (23). Around 38% of children under age 5 years are stunted; 21% are wasted; and 36% are underweight (19). The ICDS program in reducing the undernourishment among children over the past decade has been modest and slow in India than what has been reached in other countries with comparable socio-economic measure. Therefore, this study aims to identify the district level clustering of the utilization of ICDS services in India, and the present research also tried to relate it with socio-economic and demographic factors.

## Data and Methods

The study utilized the data for the present evaluation were taken from the recent round of National Family Health Survey-4 (NFHS-4) conducted in 2015–16 (19). The survey design is a stratified two stage sampling for the selection of sample and covered 29 states, 7 union territories and 640 districts (19). NFHS-4 collected 699,686 sample of ever-married women from 601,509 households. The study used kids file for the analysis, and restrict the sample (*N* = 295,622) to the children aged below 6 year during 5 year preceding the survey.

### Outcome Variable

The outcome variable for the analysis was children who received benefits from Integrated Child Development Scheme (ICDS). The question was asked to the women “During last 12 months, has [Name] received any benefits from the anganwadi or ICDS center?” Any benefits such as supplementary food, growth monitoring, immunizations, health checkups or education. The response was dichotomous 1 “Yes (received benefits from ICDS)” and 0 “No (not receive)”.

### Predictor Variable

These includes women's education, household's wealth index, place of residence, caste, religion, and owning BPL card. The education level of women was divided into two categories as no schooling and educated. Wealth quintile of households was grouped as poor (poorest and poorer) and non-poor (middle, richer, and richest) (24). Place of residence was given as rural and urban in the survey (19). Caste was grouped as scheduled caste/scheduled tribe (SC/ST) and non-scheduled caste/scheduled tribe (SC/ST) (25). Religion was categorized as Hindu and non-Hindu. Owning below poverty line (BPL) card coded as yes and no (26).

### Statistical Analysis

Bivariate and multivariate logistic regression analysis was used to analyze the data. In multivariate logistic regression analysis, we have taken those predictors which have *p*-value <0.2. Additionally, for spatial analysis in terms of utilization of ICDS in India, univariate and bivariate Moran's I index measurements were used along with the usage of spatial regression models (27). Spatial auto-correlation is being measured by using Moran's I statistics. Spatial autocorrelation represents the extent to which data points are similar or dissimilar to their spatial neighbors (28–30).

Univariate Moran's I measure the spatial auto-correlation of neighborhood values around a specific spatial location. It determines the extent of spatial non-stationary and clustering present in the data. Bivariate Moran's I examine the local correlation between an outcome variable and certain characteristics of the region. While both univariate and bivariate Moran's I aim to measure similarities and dissimilarities of spatial data, they are found to be less useful in case of uneven spatial clustering (27, 30). The formula to calculate the Moran's *I* statistic is as follows:


$$\begin{array}{l} \text{Univariate Moran'}s\text{ I }=\;\frac{n}{S_{O}}\times \frac{\Sigma_{i}\Sigma_{j}W_{ij}\left(x_{i}-\bar{X}\right)\left[x_{j}-\bar{X}\right]}{{\Sigma_{i}\left[x_{i}-\;\bar{X}\right]}^{2}} \end{array}$$

Where x is the variable of interest and $\bar{X}$ is the mean of x; n is the number of spatial units; *W*_*ij*_is the standardized weight matrix between observation i and j with zeroes on the diagonal; and *S*_*O*_ is the aggregate of all spatial weights, i.e., *S*_*O*_ = Σ_*i*_Σ_*j*_*W*_*ij*_


$$\begin{array}{l} \text{Bivariate Moran'}s\text{ I }=\;\frac{n}{S_{O}}\times \frac{\Sigma_{i}\Sigma_{j}W_{ij}\left(x_{i}-\bar{X}\right)\left[Y_{j}-\bar{Y}\right]}{{\Sigma_{i}\left[y_{i}-\;\bar{Y}\right]}^{2}} \end{array}$$

Where x and y are the variables of interest; $\bar{X}$ is the mean of x; $\bar{Y}$ is the mean of y; n is the number of spatial units; *W*_*ij*_is the standardized weight matrix between observation i and j with zeroes on the diagonal; and *S*_*O*_ is the aggregate of all spatial weights, i.e., *S*_*O*_ = Σ_*i*_Σ_*j*_*W*_*ij*_.

Value of Moran's- I range from −1 (indicating perfect dispersion) to +1 (perfect correlation). A zero value indicates a random spatial pattern. Negative (positive) values indicate a negative (positive) spatial autocorrelation. Positive autocorrelation indicates that points with similar attribute values are closely distributed in space, whereas negative spatial autocorrelation indicates that closely associated points are more dissimilar (27–31).

Univariate LISA calculates the spatial-correlation of neighborhood values around the specific spatial location (30). It determines the extent of spatial randomness and clustering present in the data. The measure [*I*_*i*_] is given by the following:


$$\begin{array}{l} \text{Univariate LISA}:\;I_{i}\;=\;\frac{n.\;\left[x_{i}-\bar{X}\right]}{\Sigma_{i}{\left[x_{i}-\bar{X}\right]}^{2}}\Sigma_{j}w_{ij}\left[x_{j}-\;\bar{X}\right] \end{array}$$

Bivariate Local Indicator of Spatial Association (LISA) measures was estimated to analyze the association of certain characteristics of regions with ICDS utilization. The bivariate LISA presented as below:


$$\begin{array}{l} \text{Bivariate LISA}:\;I_{i}\;=\frac{n.\;\left[x_{i}-\bar{X}\right]}{\Sigma_{i}{\left[y_{i}-\bar{Y}\right]}^{2}}\Sigma_{j}w_{ij}\left[y_{i}-\;\bar{Y}\right] \end{array}$$

Four types of spatial auto-correlation were generated based on the four quadrants of Moran's I scatter plots which are defined as follows:

- **Hot Spots**: districts with high values, with similar neighbors (High-High).
- **Cold Spots**: districts with low values, with similar neighbors (Low-Low).
- **Spatial Outliers**: districts with high values, but with low-value neighbors (High-Low) and districts with low values, but with higher values of neighbors (Low-High).

The spatial weights W_ij_ are non-zero when i and j are neighbors, else it remains zero (28, 29). The weight used in the analysis is Queen Contiguity weights which represents whether spatial units share the boundary or not. If the set of boundary points of unit I is denoted by the band (i), then the Queen Contiguity Weight is defined by:


$$\begin{array}{l} {\text{W}}_{\text{ij}}=\{\begin{array}{c} 1\;,bnd\;\left(i\right)\cap bnd\;\left(j\right)\neq \;\emptyset \\ 0\;,\;bnd\;\left(i\right)\cap bnd\;\left(j\right)\neq \;\emptyset \end{array} \end{array}$$

However, this allows the possibility that spatial units share only a single boundary point (such as a shared corner point on a grid of spatial units). Hence a stronger condition is to require that some *positive* portion of their boundary be shared.

In order to determine the significant correlates of ICDS service utilization in India, a set of regression models had been used. The spatial ordinary least square (OLS) regression model was used to see the extent of autocorrelation in the error term. Since the OLS confirmed spatial autocorrelation in its error term for the dependent variable, we further estimated the spatial lag model (SLM) and spatial error model (SEM) (27, 28). The underlying assumption of a spatial lag model is that the observations of the outcome variable are affected in the neighborhood areas whereas the spatial error model is used to consider the effect of those variables which are absent in the regression model but had an effect on the outcome variable. The basic difference between the two models is that the spatial lag model unlike the spatial error model does not consider the spatial dependence of the error term.

The basic equation for OLS is as follows:


$$\begin{array}{l} Y=\alpha +BX+\;\varepsilon \end{array}$$

Where Y is the outcome variable, X is the vector of predictor variables and α is the model intercept and β is the corresponding coefficient vector.

The spatial lag model suggests that the units are spatially dependent on each other and lagging to each in the nearby spatial locations (27, 30). A typical spatial lag model can be written as follows:


$$\begin{array}{l} Y_{i}=\delta \underset{j\neq 1}{\sum }W_{ij}Y_{j}+\beta X_{j}+\varepsilon_{j}\; \end{array}$$

Here *Y*_*i*_ denotes the ICDS service utilization for the *i*^*th*^ district, δ is the spatial autoregressive coefficient, *W*_*ij*_ denotes the spatial weight of proximity between district i and j, *Y*_*j*_ is the ICDS service utilization in the *j*^*th*^ district, β_*j*_ denotes the coefficient, *X*_*j*_ is the predictor variable and εj is the residual.

The spatial error model, on the other hand, considers the contribution of omitted variables that are not included in the model but can have a significant effect in the analysis (30). A Spatial Error Model (SEM) is expressed as follows:


$$\begin{array}{l} Y_{i}=\beta X_{j}+\lambda \underset{j\neq 1}{\sum }W_{ij}Y_{j}\varepsilon_{j}+\varepsilon_{i} \end{array}$$

Here, *Y*_*i*_ denotes the ICDS service utilization for the *i*^*th*^ district, λ is the spatial autoregressive coefficient, *W*_*ij*_ denotes the spatial weight of proximity between district i and j, *Y*_*j*_ is ICDS service utilization in the *j*^*th*^ district, β_*j*_ denotes the coefficient, *X*_*j*_ is the predictor variable and ε_*i*_ is the residual.

## Results

Table 1 depicts the Socio-demographic profile of study population in India. As per National Family Health Survey estimates, about 54% children below 6 years received benefits from Integrated Child Development Scheme (ICDS). Nearly 31% of respondents were having no schooling, 47% were poor, and majority of study population lived in rural areas (71.5%). Around one-third of respondents belonged to scheduled caste/scheduled tribe, 78.6% were Hindu, and about 40% of respondents belonged to below poverty line (BPL) card.

**Table 1:** Socio-demographic profile of the study population in India, 2015–16.

**Variables**	***N* = 295,646**
	**[*n* (weighted %)]**
**Benefits Received from ICDS**	
No	136,603 (45.9)
Yes	159,019 (54.1)
**Educational level**	
No schooling	93,897 (30.6)
Educated	201,749 (69.4)
**Wealth index**	
Poor	147,166 (46.9)
Non-poor	148,480 (53.1)
**Place of residence**	
Urban	70,723 (28.5)
Rural	224,923 (71.5)
**Caste**	
Scheduled caste/scheduled tribe	114,875 (31.9)
Non-scheduled caste/scheduled tribe	180,771 (68.1)
**Religion**	
Hindu	213,390 (78.6)
Non-Hindu	82,256 (21.4)
**Has BPL card**	
No	182,804 (60.8)
Yes	112,842 (39.2)

Bivariate and estimates from logistic regression analysis for children who received benefits from ICDS were presented in Table 2. Children whose mothers are educated, 1.42 times more likely to receive benefits from ICDS. Children belonged to poor and rural areas were 1.03 and 1.81 times significantly more likely to receive benefits from ICDS, respectively, compared to their counterparts. Moreover, scheduled caste/schedules tribe and children below poverty line were 1.27 and 1.47 times more likely to receive benefits from ICDS, respectively, compared to Non-SC/ST children and those who do not have BPL card.

**Table 2:** Results from bivariate and logistic regression analysis for children who received benefits from ICDS by background factors in India, 2015–16.

**Variables**	**ICDS (%)**	**OR (95% C.I.)**
**Educational level**		
No schooling	51.6	Ref.
Educated	55.2	1.42*** (1.40–1.44)
**Wealth index**		
Poor	58.1	1.03*** (1.01–1.05)
Non-poor	50.5	Ref.
**Place of residence**		
Urban	40.2	Ref.
Rural	59.6	1.81*** (1.77–1.84)
**Caste**		
Scheduled caste/scheduled tribe	61.1	1.27*** (1.25–1.29)
Non-scheduled caste/scheduled tribe	50.8	Ref.
**Religion**		
Hindu	55.5	Ref.
Non-Hindu	48.7	0.72*** (0.7–0.73)
**Has BPL card**		
No	49.3	Ref.
Yes	61.6	1.47*** (1.45–1.49)

*Ref, Reference; OR, Odds ratio; CI, Confidence Interval; BPL, Below Poverty Line; ICDS, Integrated Child Development Scheme.*

***p < 0.001.

Table 3 depicts the values of univariate and bivariate Moran's I statistics in India. Univariate Moran's I statistics represent the spatial auto-correlation of outcome and predictor variables. The value of spatial-autocorrelation for benefits received from ICDS was 0.61 which means a high dependence of the outcome variable over districts of India. Moreover, highest Moran's I value among predictors were witnessed by children from poor wealth quintile (0.74) followed by children belonged to Hindu religion (0.72), and who had BPL card (0.71). Additionally, table reveals that spatial autocorrelation between children who received benefits from ICDS and those who had BPL card was 0.27, and among children who belonged to Hindu religion was 0.25.

**Table 3:** Univariate and Bivariate Moran's I Values for outcome and predictors in India, 2015–16.

**Variables**	**Univariate**	**Bivariate**
		**ICDS**
Child received benefits from ICDS (%)	0.61 (0.001)	-
Educated (%)	0.68 (0.001)	0.19 (0.001)
Poor wealth quintile (%)	0.74 (0.001)	0.03 (0.042)
Rural place of residence (%)	0.42 (0.001)	0.09 (0.001)
Scheduled caste/scheduled tribe (%)	0.57 (0.001)	0.06 (0.001)
Hindu (%)	0.72 (0.001)	0.25 (0.001)
Has BPL (%)	0.71 (0.001)	0.27 (0.001)

Table 4 provides estimates for spatial regression estimates for children who received benefits from ICDS and its predictors for 640 districts of India. From the OLS estimates, it was confirmed that education (β: 0.407, *p* < 0.001), rural place of residence (β: 0.22, *p* < 0.001), schedules caste/scheduled tribe (β: 0.098, *p* < 0.001), Hindu religion (β: 0.196, *p* < 0.001), and those had BPL card (β: 0.245, *p* < 0.001) were found to be significant spatial predictors of children who received benefits from ICDS in India. The value of adjusted *R*^2^ was 0.40 and the value for AIC was found to be 5,269.

**Table 4:** Spatial regression model for estimating spatial association between benefits received from ICDS and background factors in India, 2015–16.

**Variables**	**OLS (*p*-value)**	**SLM (*p*-value)**	**SEM (*p*-value)**
Educated (%)	0.407 (0.001)	0.26 (0.001)	0.291 (0.000)
Poor wealth quintile (%)	0.009 (0.800)	−0.018 (0.532)	0.013 (0.765)
Rural place of residence (%)	0.22 (0.001)	0.20 (0.001)	0.230 (0.000)
Scheduled caste/scheduled tribe (%)	0.098 (0.001)	0.062 (0.006)	0.084 (0.003)
Hindu (%)	0.196 (0.001)	0.11 (0.001)	0.122 (0.000)
Has BPL card (%)	0.245 (0.001)	0.13 (0.001)	0.167 (0.000)
***N*** **(Sample)**	640	640	640
**Rho**		0.54 (0.000)	
**Lambda**			0.74 (0.000)
**AIC**	5,269.4	5,026	4,942
**Adjusted R**	0.40	0.75	0.69

*AIC, Akaike information criterion; OLS, Ordinary least square; SLM, Spatial lag model; SEM, Spatial error model*.

The value of lag coefficient was 0.54 (*p* < 0.001) from the SLM which signifies that a change in the children who received benefits from ICDS in a particular district may statistically lag the rate of ICDS coverage by 54% in the neighboring districts. In the spatial lag model, it was found that education (β: 0.26, *p* < 0.001), rural place of residence (β: 0.20, *p* < 0.001), Hindu (β: 0.11, *p* < 0.001), and had BPL card (β: 0.13, *p* < 0.001) were significantly associated with ICDS coverage in India. The respective model splits the value of adjusted *R*^2^ as 0.75 and value for AIC was found as 5,026.

However, as per the theory of spatial regression models, the model with the lowest AIC value and highest *R*^2^-value is considered to be the best fit model. Therefore, as per our model estimates the lowest AIC and highest adjusted *R*^2^-value was found to be of spatial error model (SEM) which makes it the best fit model among all the three models. The spatial error model was having an AIC value of 4,942 and an adjusted *R*^2^-value of 0.69. Interestingly the value of Lambda (spatial autoregressive coefficient)/error lag value was 0.74 (*p* < 0.001) which signifies that spatial influence on ICDS coverage through the omitted variables not present in the SEM.

The model reveals that if in a district educated women increase by 10% then the children received benefits from ICDS get increased by 2.9%. Similarly, if in a district children lived in rural areas and belonged to Hindu religion by 10% then children who received benefits from ICDS get increased by 2.3 and 1.2%, respectively. Additionally, if in a district children had BPL card increase by 10% then ICDS coverage get increased by 1.6%. Moreover, mother's education (β: 0.291, *p* > 0.001), rural place of residence (β: 0.230, *p* > 0.001), Scheduled caste/Scheduled tribe status residence (β: 0.084, *p* > 0.05), Hindu religion (β: 0.122, *p* > 0.001), and having BPL card (β: 0.167, *p* > 0.001) were positively associated with children who received benefits from ICDS. The results simply imply that districts with a higher percentage of children whose mother were educated and lived in rural areas, belonged to Hindu religion and having BPL card had higher chances to get benefited from the ICDS.

### Spatial Analysis

**Figure 1:**
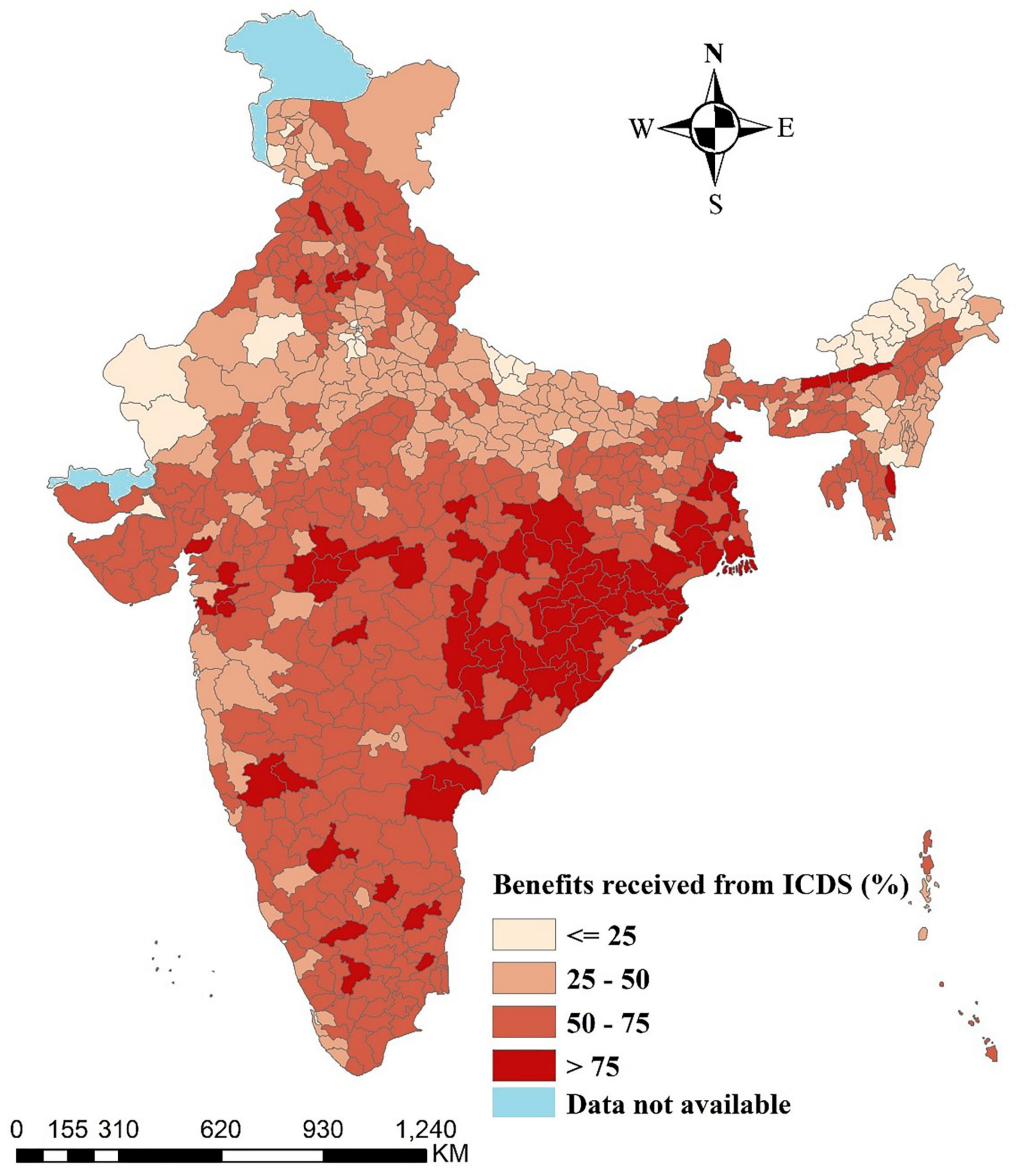
Percentage distribution of children who received benefits from ICDS.

Figure 1 displays the percentage distribution of children who received benefits from ICDS across the districts of India. The color pattern reveals that spatial differences in the ICDS service utilization among children aged below 6 years. Moreover, deeper color indicates higher utilization of ICDS services and light color indicates the lower coverage of ICDS among children. More than 75% of the children who received benefits from ICDS belonged in the districts of Odisha (26), Chhattisgarh (13), and West Bengal (10) and few districts of Madhya Pradesh and Maharashtra.

#### Univariate LISA

**Figure 2:**
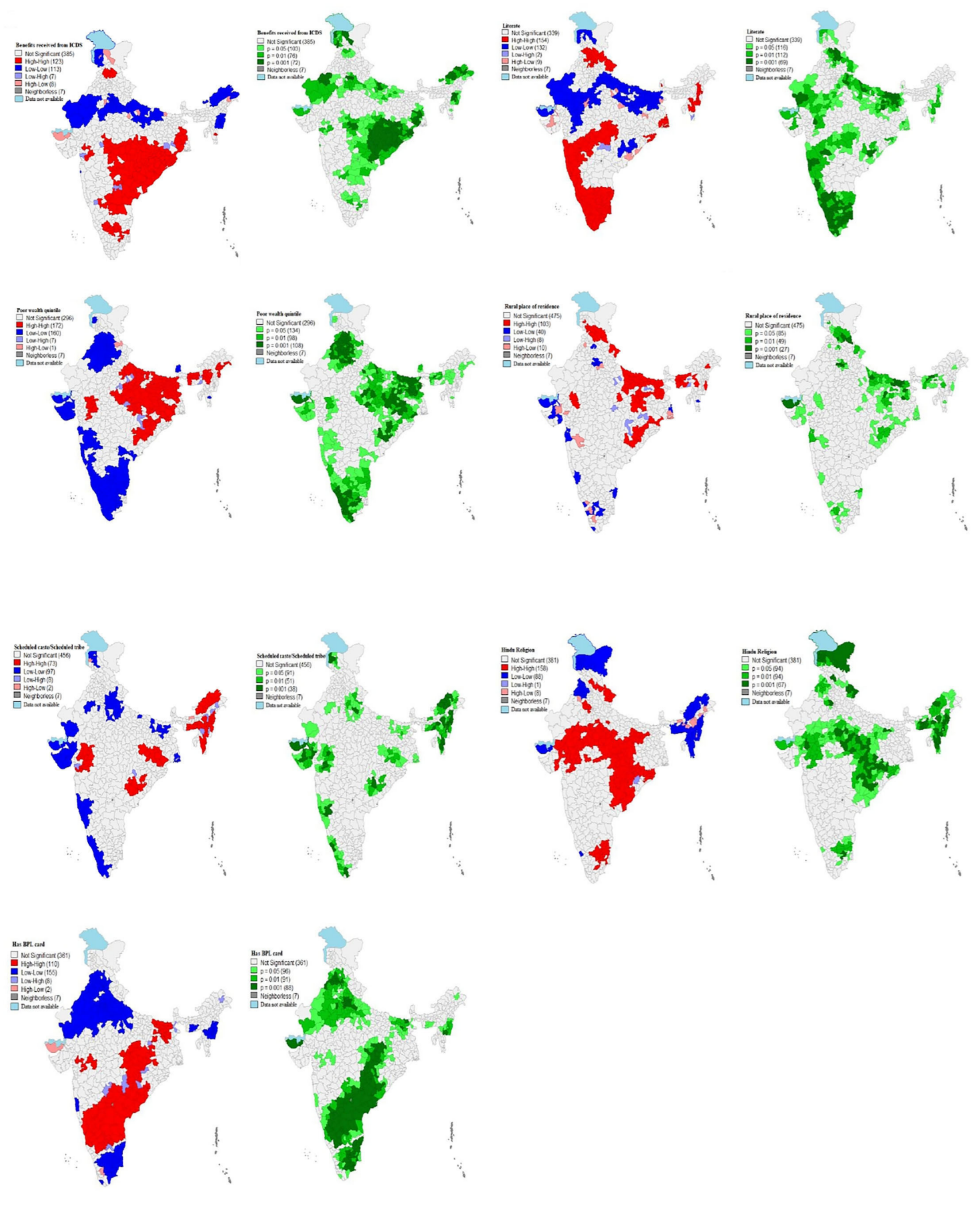
Univariate Local Indicator of Spatial Association (LISA) (cluster and significance) maps for outcome and independent variables for districts of India, 2015–16.

Figure 2 represents univariate LISA (cluster and significance) maps for outcome and independent variables for the districts of India. The Moran's I statistic was 0.61, suggested a highly significant spatial dependence in the ICDS service utilization in India. A total of 123 districts from Odisha, Chhattisgarh, Jharkhand and some parts of Madhya Pradesh, West Bengal, Andhra Pradesh and Maharashtra formed the hot spots (high-high) while 113 districts from Rajasthan, Uttar Pradesh, Bihar, Arunachal Pradesh and Manipur formed the cold spots (low-low) of ICDS service utilization (Moran's I = 0.61, *p*-value = 0.001) across India. A total of 15 districts have been found as spatial outliers (high-low or low-high) of ICDS services utilization in the country.

Univariate LISA cluster map of literate mothers (Moran's I = 0.68, *p*-value = 0.001) identified 154 “hotspots” and 132 “cold spots”. Most of the hotspots are located in Tamil Nadu, Kerala, Goa and parts of Maharashtra, Telangana, Uttarakhand, Himachal Pradesh and north-east states (Mizoram, Manipur, Assam). Whereas, it is lower in the states of Rajasthan, Uttar Pradesh (UP), Bihar and some parts of Gujrat, Odisha, and Madhya Pradesh (MP).

A total of 172 districts from Uttar Pradesh, Bihar, Odisha, West Bengal, Jharkhand and some parts of Madhya Pradesh, Assam, Arunachal Pradesh and Maharashtra formed the hot spots (high-high) while 160 districts from Rajasthan, Uttar Pradesh, Bihar, Arunachal Pradesh and Manipur formed the cold spots (low-low) of poor wealth quintile children across India. A total of 8 districts have been found as spatial outliers (high-low or low-high) of poor wealth quintile child in the country.

Univariate LISA cluster map of rural resident children (Moran's I = 0.42, *p*-value = 0.001) identified 103 “hotspots” and 40 “cold spots”. Most of the hotspots are located in Himachal Pradesh, Bihar, Jharkhand and parts of Uttar Pradesh (UP), Odisha, Uttarakhand, and Rajasthan Whereas, it is lower in some parts of state like Gujrat, Delhi, Maharashtra, Karnataka, and Tamil Nadu.

A total of 73 districts from North-east states (Arunachal Pradesh, Nagaland, Assam, Mizoram), Jharkhand and some parts of Odisha, Gujrat and Maharashtra formed the hot spots (high-high) while 97 districts from Gujrat, Kerala, Bihar, and some parts of Uttar Pradesh, Rajasthan, Maharashtra, Karnataka, Jammu & Kashmir (J&K) formed the cold spots (low-low) of Schedule caste/Schedule tribe children (Moran's I = 0.61, *p*-value = 0.001) across India. A total of 10 districts have been found as spatial outliers (high-low or low-high) of Schedule caste/Schedule tribe children in the country.

Univariate LISA cluster map of Hindu children (Moran's I = 0.72, *p*-value = 0.001) identified 103 “hotspots” and 40 “cold spots”. Most of the hotspots are located in Uttarakhand, Jharkhand, Chhattisgarh, Odisha, Madhya Pradesh (MP) and parts of Delhi, Tamil Nadu, Gujrat, Rajasthan and Uttar Pradesh (UP) Whereas, it is lower in the states of Jammu & Kashmir, Punjab, North-east states and some parts of Gujrat and Kerala.

A total of 110 districts from Southern states (Karnataka, Andhra Pradesh, Telangana), Bihar and some parts of Jharkhand, Chhattisgarh and Madhya Pradesh (MP) formed the hot spots (high-high) while 155 districts from Rajasthan, Punjab, Uttar Pradesh (UP), Delhi, Tamil Nadu and some parts of Maharashtra, Manipur and Meghalaya formed the cold spots (low-low) of BPL card children family (Moran's I = 0.71, *p*-value = 0.001) across India.

#### Bivariate LISA

**Figure 3:**
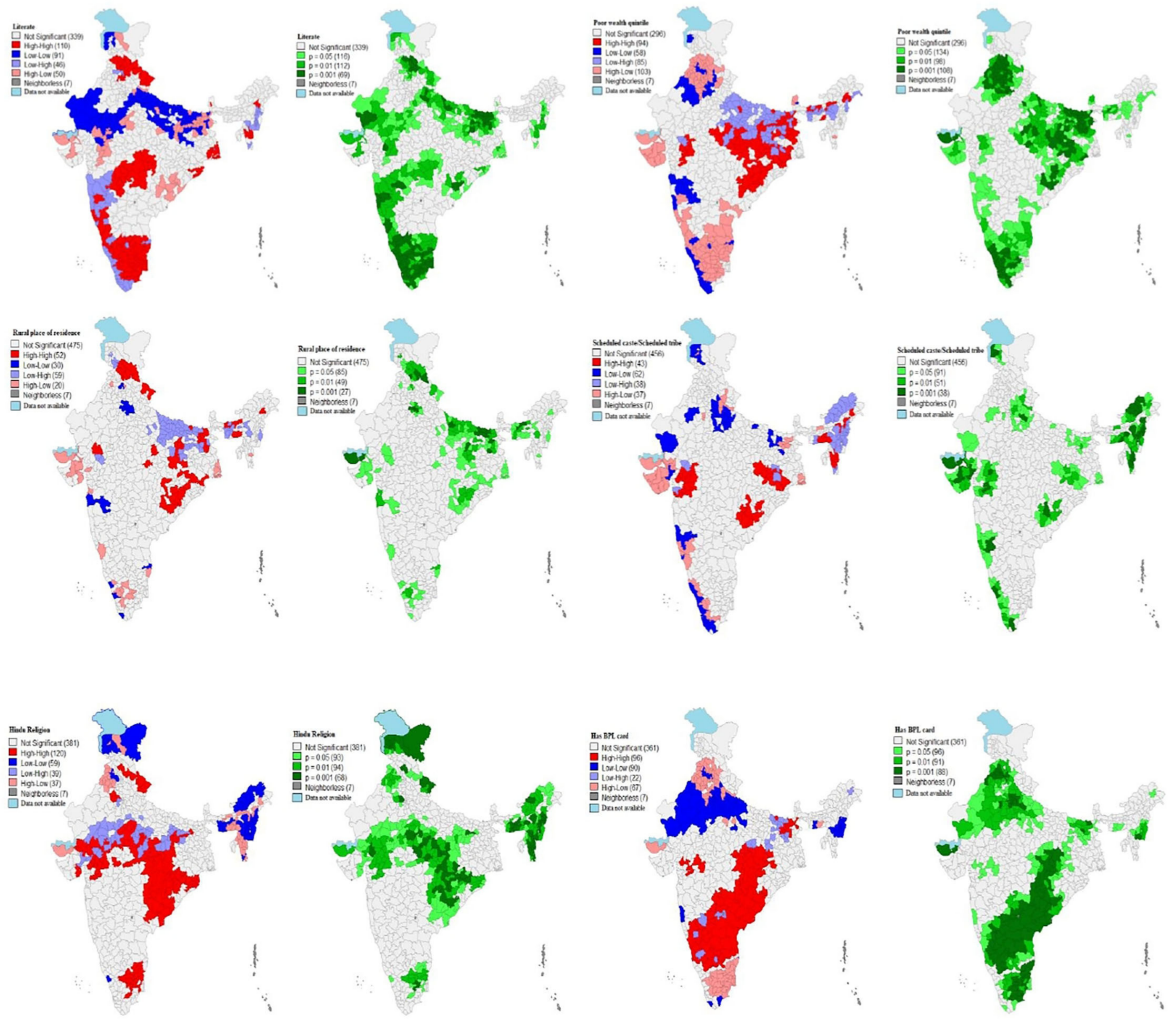
Bivariate Local Indicator of Spatial Association (BiLISA) (cluster and significance) maps for outcome vs. predictor variables for districts of India, 2015–16.

Figure 3 displays bivariate LISA (cluster and corresponding significance) maps for understanding the spatial association of ICDS services utilization with independent variables for the districts of India.

Out of 640 districts, 110 districts were classified as hot spots (defined as high ICDS services utilization and high literate mothers) from the states of Tamil Nadu and some districts of Karnataka, Maharashtra, Madhya Pradesh (MP), Himachal Pradesh, Uttarakhand, West Bengal, Odisha and Mizoram. while 91 districts from Rajasthan, Uttar Pradesh and some parts of Bihar and Jammu & Kashmir (J&K) formed the cold spots (defined as low ICDS services utilization and low literate mothers) across India. A total of 96 districts have been found as spatial outliers (high-low or low-high) of ICDS services utilization and literate mothers in the country.

A total of 94 districts from Odisha, Jharkhand and some parts of Madhya Pradesh, Assam, Bihar, Chhattisgarh and Maharashtra formed the hot spots (defined as high ICDS services utilization and poor wealth quintile children) while 58 districts from Kerala and parts of Rajasthan, Maharashtra and Delhi formed the cold spots (low-low) of ICDS services utilization and poor wealth quintile children across country.

LISA map (Figure 3) which indicated that about 52 of 640 districts had highest prevalence of ICDS services utilization and highest rural place resident child. These districts were mostly from the states of Himachal Pradesh and some parts of Uttarakhand, Odisha, Uttar Pradesh, Bihar, Jharkhand, Meghalaya, Assam, and Rajasthan. A total of 30 districts were classified as cold spots (defined as low ICDS services utilization and low level of rural resident children) from some districts of Maharashtra, Kerala, and Tamil Nadu.

A total of 43 districts from Jharkhand, Mizoram and some parts of Gujrat, Odisha, West Bengal, Meghalaya and Arunachal formed the hot spots (defined as high ICDS services utilization and high level of SC/ST children) while 62 districts from Kerala and parts of Rajasthan, Uttar Pradesh, Bihar and Maharashtra formed the cold spots (low-low) of ICDS services utilization and SC/ST children across country.

A total of 120 districts from Chhattisgarh, Jharkhand, Uttarakhand and some parts of Madhya Pradesh, Andhra Pradesh, Odisha, Gujrat and Tamil Nadu formed the hot spots (defined as high ICDS services utilization and high Hindus children) while 59 districts from Jammu & Kashmir (J&K), Arunachal Pradesh, Manipur, Nagaland and parts of Meghalaya and Punjab formed the cold spots (low-low) of ICDS services utilization and Hindu religion children across country.

Out of 640 districts, 96 districts were classified as hot spots (defined as high ICDS services utilization and high level of BPL card child family) from southern states (Karnataka, Andhra Pradesh and Telangana), Chhattisgarh and some districts of Gujrat, Jharkhand, Bihar and Madhya Pradesh (MP). While 90 districts from Rajasthan, Uttar Pradesh, Manipur and some parts of Maharashtra, Punjab and Meghalaya formed the cold spots (defined as high ICDS services utilization and low BPL card children family) of poor wealth quintile children across India.

## Discussion

The study have highlighted utilization of the ICDS services coupled with socio-economic and demographic dynamics. The spatial clusters have depicted the utilization patterns in the country and the associated factors along with it. The benefits of ICDS was majorly received by the children belonging to districts of Odisha, West Bengal and some districts of Madhya Pradesh and Maharashtra. On the other hand, the districts of populous state like Uttar Pradesh where the health indicators are the poorest, have least utilization of the benefits of ICDS. This could be owing to the inadequate distribution of funds across states along with poor governance (32). The utilization was significantly higher in the rural areas than urban residences. But still studies have shown that a significant proportion of women in rural areas and children did not receive ICDS and hence the concern regarding the design and implementation of the program (6, 33). The regional accessibility of the AWCs and the rural transport facilities for the mother and children are essential factors. For instance, the Wardha district in Maharashtra has only 42% of the households having AWCs in a kilometer range (34).

The findings have also highlighted the socio-cultural factors that shape the utilization patterns in a region and neighboring areas too. The ones belonging to the scheduled caste or tribe and Hindu religion were the significant beneficiaries. The poor families possessing BPL cards also received the benefits from ICDS. Hence, the economically better and non-Hindu people are observed to have lesser utilization. The understanding behind the reasons for not opting the benefits are very less explored and reliance on the services provided plays an important part (34, 35). Education is another significant factor found from the results where utilization was more among the ones with educated mothers. Studies have also observed a positive association between education of mother and availing the ICDS preschool services for children (36, 37). This relation can also be corroborated by the studies that had highlighted the correlation of basic education and awareness about services (38). This result suggests promoting education of women plays a critical role in raising awareness about service utilization (35).

The service utilization was found to be more clustered in the Southern states with high level of possession of BPL cards and Hindu religion. A study based on Tamil Nadu had reported that among the caste groups the poorest among the scheduled castes had utilized the ICDS services the least (39). In southern part, the BPL card possession was high as reported by a study based on South India, where the services utilization was maximum for JSSK (100%) followed by ICDS (97.9%) (40). A study by Davey et al. (41), showed that due to lack of easy accessibility of AWC, unavailability of space at the AWC and poor quality of distributed food were some of the major reasons of non-utilization of services (41). Also, mothers felt difficulty in carrying the child to the AWCs and with not enough contact with the AWC workers makes it more difficult (20).

One of the important aspects of the ICDS services is to reduce child malnutrition and mortality with adequate maternal and child healthcare (37, 42). Hence, there needs to be a strengthened relation between utilization of services by the mothers and their children. National and international studies have also recommended comprehensive strategies for the continuum of services in the Reproductive, Maternal, New-born and Child healthcare (RMNCH) (43, 44). The strategies include the time dimension for capturing the continuum of care factor. Maternal and child health is unfortunately not captured in any longitudinal data and hence studies used the NFHS to understand RMNCH at district level in India (45).

The ICDS has a wide range of services that can ensure continuum of mother and child health as well as improve utilization in the regions lagging behind services of ICDS. The use of technology can enhance tracking of the progress of pregnant women and children, and increasing the service utilization rates. There are studies that also suggested the tracking of the prospective mothers and their children by working on community levels with the health workers (45, 46).

The study though provides a details spatial analysis and explores the socio-economic and demographic factors, there exists certain limitations. The disaggregated data on certain services of ICDS such as take home ration for 0–35 months old children and AWC meals for the children of 36–72 months age, was not available. But the study has used the data available on all the other services provided to give holistic scenario. A major limitation can be considered regarding NFHS being a cross-sectional data that doesn't necessarily conclude on the causality between the outcome and its correlates.

## Conclusion

The present study is an essential addition to literature on the utilization of ICDS services that explores the spatial patterns with socio-economic and demographic factors. This is the first-ever study that examined the complex interplay of the rural population, female illiteracy, poverty, SC/ST population, and Hindu population with non-utilization of ICDS services among children in the district in India. The study highlights the inter-district geographical disparities in the non-utilization of ICDS services. Further, it confirms that underprivileged districts in terms of the rural population and poor households are also disadvantageous in the utilization of ICDS services. The results indicates the scope for improvisation of the services in the backward states and districts like that of Uttar Pradesh. More awareness regarding the ICDS services are suggestive as maternal education has come out to be a significant factor. Further education of holistic development of children and capacity building of AWWs are also critical. However, future studies on community level with qualitative analysis is recommended to obtain a depiction of what is happening at ground level.

## Data Availability Statement

Publicly available datasets were analyzed in this study. This data can be found here: https://dhsprogram.com/data/available-datasets.cfm.

## Author Contributions

The concept was drafted by PK, SK, and RB. All authors contributed to the analysis design, comprehensive writing of the article, and read and approved the final manuscript.

## Conflict of Interest

The authors declare that the research was conducted in the absence of any commercial or financial relationships that could be construed as a potential conflict of interest.

## Publisher's Note

All claims expressed in this article are solely those of the authors and do not necessarily represent those of their affiliated organizations, or those of the publisher, the editors and the reviewers. Any product that may be evaluated in this article, or claim that may be made by its manufacturer, is not guaranteed or endorsed by the publisher.
